# An authorizable and preprocessable data transmission scheme based on elliptic curves

**DOI:** 10.1038/s41598-025-05822-3

**Published:** 2025-07-01

**Authors:** Zhongshan Zhu, Liutao Zhao, Yong Pan, Xiaojuan Lu, Zheng Wang, Fanyin Meng

**Affiliations:** 1 Beijing Computing Center Co.,Ltd., Beijing, 100094 China; 2 Beijing Academy of Science and Technology, Beijing, 100089 China; 3 Beijing Beike Rongzhi Cloud Computing Technology Co., Ltd., Beijing, 100094 China

**Keywords:** Secure transmission, Preprocessable, Authorization signature, Applied mathematics, Computational science, Computer science, Scientific data

## Abstract

Current data security transmission schemes are based on the idea of signcryption, where the sender performs encryption and generates signatures within a single logical step. However, on one hand, the complexity of signing is relatively low, while the encryption and communication complexities for large amounts of data are high, leading to low overall transmission efficiency. On the other hand, once the receiver obtains the ciphertext, they can decrypt it, reducing the sender’s control over the data. Therefore, this paper proposes a data security transmission scheme that supports authorization and preprocessing. The scheme first preprocesses the computationally expensive data encryption and transmission operations, and then performs the authorization signature to improve efficiency. Specifically, based on the *R* value from Schnorr’s signature, the sender’s public key, and the receiver’s public key, a one-time public key is computed and used to encrypt the data before sending it to the receiver. The receiver can compute the corresponding one-time private key to decrypt and obtain the plaintext data, but only after receiving the *s* value from the sender’s Schnorr signature. Additionally, before the authorization signature *s* is published, the receiver cannot decrypt the data, ensuring both authorization unforgeability and data confidentiality, while also enhancing the sender’s control over the decryption timing. Experimental results show that for a 1KB data transmission, the execution times for the one-time public key generation algorithm, encryption algorithm, authorization algorithm, decryption algorithm, and signature verification algorithm were 3.34/28.37/0.58/3.32/4.58 ms, respectively, indicating high efficiency for each algorithm. Comparison tests show that for data sizes ranging from 50K to 1600K, using the preprocessing method can reduce execution time by about 68%.

## Introduction

In today’s data-driven economy, data becomes a critical factor of production, and the ability to effectively manage and utilize data resources serves as a key indicator of a nation’s competitiveness^1^. Government agencies, enterprises, and academic researchers enhance decision-making efficiency, optimize service customization, and drive scientific and technological progress through data collection and analysis^2,3^. Data transmission and sharing refer to the process of transferring data stored in information systems of enterprises and governments to data recipients for analysis and use, according to agreed rules and protocols between data providers and data demanders^4^. Through data transmission and sharing, data is separated from its owner and transferred from its original application scenario to a more targeted one, realizing its value transformation. The importance of secure data transmission and sharing is increasingly prominent, and ensuring security and confidentiality during data transmission is essential for maintaining personal privacy, commercial secrets, and national security.

Secure data transmission primarily faces two core issues: data confidentiality and data integrity^5^. Confidentiality aims to prevent unauthorized parties from accessing or misusing data during transmission, while integrity ensures that data remains unaltered during transmission. Solving these two issues mainly relies on cryptographic encryption algorithms and digital signature algorithms^6^. Traditional solutions typically adopt the sign-then-encrypt strategy, where data is first digitally signed and then encrypted to ensure secure transmission over an insecure network. This approach requires encryption and signature functions to be implemented in two separate logical steps, resulting in low execution efficiency and high costs. Scholars propose the data transmission scheme called signcryption^7^, which, compared to the traditional ”sign-then-encrypt” method, cleverly integrates encryption and digital signature functions into a single logical step. This significantly reduces the amount of mathematical operations, thereby improving the overall efficiency of data transmission and effectively reducing computational overhead and communication costs. However, in the signcryption scheme, when the receiver receives the ciphertext, they can directly decrypt and use the data, which prevents the sender from controlling how the receiver processes and uses the data. In some legal frameworks, such as the EU General Data Protection Regulation (GDPR), the sender may be required to be responsible for the entire lifecycle of the data. In this case, the sender loses control, potentially facing compliance risks^8^.

From signcryption schemes based on bilinear pairing^9–11^ to signcryption schemes based on RSA^12–14^, and then to signcryption schemes based on elliptic curve cryptography (ECC)^15–18^, researchers continually optimize data transmission schemes in different application scenarios. such as the Internet of Vehicles system in the intelligent transportation Internet of Things^14,19–21^, smart terminal devices in cyber-physical power systems^22–24^, and electronic healthcare systems^16,25,26^, etc. However, despite significant progress in improving data transmission efficiency, these schemes still have limitations: First, digital signature and encryption operations are executed synchronously within a single step. Due to the low computational complexity of signature operations, this approach will significantly limit the efficiency of the overall solution when dealing with encryption operations with high computational complexity. This problem is particularly prominent in scenarios where large amounts of data need to be processed. Second, for data with a high level of importance, the sender has too little control over the data. The sender can only control the timing of data transmission but not the timing of data usage. To enhance the sender’s control over data, a remote authorization decryption strategy can be used. In this approach, the receiver must obtain authorization from the sender before decrypting the data, and the sender can revoke this permission at any time.

Given the above limitations,this paper proposes an authorizable and preprocessable data security transmission scheme. The main work of this paper is as follows:

- We propose an authorizable and preprocessable data security transmission scheme. The sender preprocess the encryption operation of a large amount of data, and send the ciphertext to the receiver. The receiver cannot decrypt immediately to ensure the security of the data transmission process.By asynchronously handling the encryption and signature operations, the efficiency of the data transmission scheme is improved. During decryption, the receiver have to request an authorization value from the sender. The introduction of the authorization mechanism enhance the sender’s control over the timing of the receiver’s decryption, thereby increasing the sender’s control over the data.
- The formal definition of the scheme and its specific construction are provided. The security of the scheme is proven through a specific construction example, demonstrating that it satisfies the Existential UnForgeability Under Adaptive Chosen Message Attacks (EUF-CMA) security in a game-based security mode.
- Through comparative analysis, the unique characteristics of the scheme are described in detail, including preprocessability, authorization association and authorization unforgeability.The scheme shows significant advantages in terms of computational cost and communication overhead. The experiment is conducted on a file with a size of 1KB. The execution time of the one-time public key generation algorithm, encryption algorithm, authorization algorithm, decryption algorithm and signature verification algorithm of the scheme are 3.34/28.37/0.58/3.32/4.58 ms respectively. The execution efficiency of each algorithm is high. Further, the experiment is conducted on file data with sizes ranging from 50KB to 1600KB. The results show that the preprocessing method adopted by this scheme can improve the efficiency by about 68%.

## Related works

Existing data security transmission schemes mainly rely on two types of basic cryptographic algorithms: encryption algorithms and digital signature algorithms. Encryption algorithms, such as the widely used AES (Advanced Encryption Standard)^27^ and RSA (Rivest-Shamir-Adleman) algorithm^28^, are used to ensure the confidentiality and consistency of data^29^. On the other hand, digital signature algorithms, such as DSA (Digital Signature Algorithm) and ECDSA (Elliptic Curve Digital Signature Algorithm)^30^, are used to ensure the integrity and unforgeability of data by generating a unique digital fingerprint to verify the source and content of the message. Traditional data transmission schemes adopt the sign-then-encrypt approach, and its execution process is: first digitally sign the data, and then encrypt the data to achieve secure transmission in an insecure network. However, performing encryption and digital signature operations separately is very expensive in terms of computational cost and communication overhead. In order to improve the efficiency of data transmission, Zheng proposed an efficient scheme called signcryption in 1997^7^. The core idea was to integrate the digital signature and encryption processes to improve efficiency by sharing some calculation steps. For example, in the signcryption scheme based on the discrete logarithm problem, signature and encryption could share the same random number generation process, reducing computational overhead. Zheng’s experiments demonstrated that signcryption scheme could reduce computational costs by approximately 50% and communication overhead by 85% compared to traditional schemes^7^. signcryption enabled simultaneous completion of both digital signature and public key encryption in a single logical step, ensuring the confidentiality, integrity, authentication, and non-repudiation of data transmission.

In the development of data transmission schemes based on the signcryption framework, Zheng and Imai^31^ were the first to apply elliptic curve cryptography^32^ to signcryption scheme. They proposed a new data transmission scheme based on elliptic curves, with security relying on the elliptic curve discrete logarithm problem (ECDLP)^33^. Compared to traditional ElGamal^34^ and RSA^28^ systems, this approach offers higher security and computational efficiency. However, Zheng’s scheme had a notable drawback: the signature could not be verified by a third party. To address this issue, Bao and Deng^35^ improved Zheng’s system by proposing a scheme that allows signature verification without the receiver’s private key, significantly enhancing the practicality and applicability of the system. Since then, an increasing number of data transmission schemes based on the signcryption framework have been proposed. For example, Yang et al.^9^ introduced a certificateless aggregate signcryption scheme to enable secure data transmission in vehicular ad hoc networks, addressing privacy leakage, certificate management, and key escrow issues in its security alert system. Niu et al.^10^ proposed a heterogeneous hybrid signcryption scheme to achieve secure data transmission between identity-based encryption systems and certificateless encryption systems, offering a solution for secure communication between heterogeneous cryptographic systems. Luo et al.^11^ introduced a generalized signcryption data transmission scheme, integrating both signature and signcryption modes into a single algorithm to meet the diverse security needs in complex data transmission scenarios. Although these schemes have achieved significant results in their respective application domains, they are all constructed based on bilinear pairing, which leads to high computational complexity and increases the cost of secure data transmission.

In order to reduce the computational cost of data transmission, a variety of data security transmission schemes based on non-bilinear pairing have been proposed to improve the efficiency of data transmission.Cagalaban et al.^12^ proposed an identity-based signcryption scheme for secure data transmission within wireless local area networks. The scheme was based on the RSA algorithm and combined identity authentication and data encryption to provide security guarantees for data transmission in wireless environments. To address the issues of certificate management and key escrow in Cagalaban’s scheme^12^, Liu et al.^13^ proposed a certificateless signcryption scheme based on the RSA algorithm. This scheme, tailored for secure data transmission in wireless body area networks, eliminates the need for certificate management and key escrow, overcoming the limitations of previous schemes in these aspects. Zhang et al.^14^ proposed a pairing-free, certificateless data security transmission scheme also based on the RSA algorithm. This scheme was specifically designed for secure data transmission in wireless body area networks (WBANs). By eliminating the need for certificate management and key escrow, it overcomed the limitations of previous schemes in these aspects. However, RSA algorithms face limitations in terms of key length and computational overhead. To achieve the same level of security, RSA requires longer key lengths, which increases both computational and communication costs^24^. To overcome these limitations, research has gradually shifted towards ECC-based data security transmission. ECC offers the same security with shorter key lengths and provides higher computational efficiency and storage advantages. Singh and Patro^15^ proposed an ECC-based signcryption scheme for secure data transmission in low-computation environments like Radio Frequency Identification systems, with strong resistance to interference. Mohammed et al.^16^ proposed a data security transmission scheme for electronic healthcare systems, using elliptic curve cryptography to encrypt and protect the privacy of patient medical records. Yu et al.^17^ proposed a certificateless elliptic curve aggregate signcryption scheme for Internet of Things environments, improving communication and storage efficiency while enhancing computational efficiency in the verification process. Li et al.^18^ implemented a certificateless aggregate signcryption scheme in resource-constrained cyber-physical power systems to achieve secure data transmission between devices, with the added ability to detect illegal signcryption, thereby enhancing the robustness of data security transmission.

In summary, existing data secure transmission schemes use the signcryption mechanism to improve efficiency. However, the signature and encryption operations in the signcrytion scheme still need to be executed synchronously in order, which limits the overall efficiency of the data transmission solution, especially when the encryption operation has high computational complexity. To solve this problem, this paper proposes an authorizable and preprocessable data transmission scheme based on elliptic curves, which asynchronousizes signature and encryption operations. The sender can pre-process the encryption operation and send the ciphertext, and the receiver needs to obtain the authorization value generated by the sender to decrypt and verify the signature to ensure data security. This asynchronous processing method significantly improves overall efficiency. In addition, the authorization mechanism enhances the flexibility of data transmission, allowing the sender to control the recipient’s decryption timing, which is particularly important in sensitive data distribution or time-sensitive data transmission scenarios.

## System framework

### Notations

To facilitate the understanding of our scheme, we list the main symbols and their descriptions used throughout this paper in Table 1.

**Table 1:** Meaning of scheme notations

Category	Notation	Description
Scheme construction	$\leftarrow$	Assignment operation
	$\lambda$	Security parameter
	*p*, *q*, *n*	Prime numbers
	$F_q$	Finite field with characteristic *q*
	$Z_n$	Integers modulo *n*
	$Z_n^*$	Multiplicative group modulo *n*
	*a*, *b*	Elements in $F_q$
	$(\mathbb {G},G,p)$	Cyclic group of order *p*, with generator *G*
	$(0,1)^*$	Set of all binary strings
	$\alpha _1,\alpha _2$	Random integers in $\mathbb {Z}_n$, used as private keys
	*r*, *k*	Random elements from $\mathbb {Z}_n^*$
	*i*, *j*	Index values
	*msg*	Plaintext message
	$\sigma =\left( R,s \right)$	Signature of *msg*
	$C=\left( C_1,C_2,C_3 \right)$	Ciphertext, composed of three components
	(*PK*, *sk*)	Public/private key pair. Among them, *PK* represents the public key and *sk* represents the private key.
	$(PK^{OneTime},sk^{OneTime})$	One-time public/private key pair for encryption/decryption
Complexity assumptions	*G*, *H*	Elements of group
	*a*, *b*, *c*	Integers from the set of integers
Security proof	$\mathcal {A}$	Adversary in the security model
	$\mathcal {B}$	Simulator in the security model
	$\mathcal {H}$	Random oracle
	$\varepsilon$	Advantage of the adversary
	*t*	Time complexity of the adversary
	$q_s$	Number of signing queries
	$\iota ,z,w$	Secret random values selected from $\mathbb {Z}_p^*$

### Overview

**Fig. 1:**
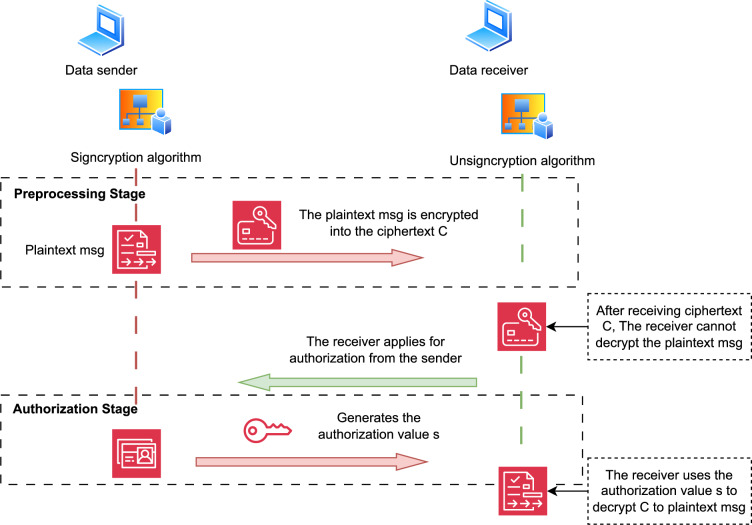
The authorizable and preprocessable data transmission scheme.

The goal of the authorizable and preprocessable data transmission scheme is to ensure the secure transmission of data while the sender preprocesses the encryption operations with higher complexity in advance, and asynchronously decrypts the encryption algorithm and the digital signature algorithm, thereby improving the efficiency of data transmission. As shown in Fig. 1, the scheme includes two participants: the data sender and the data receiver. The sender encrypts and generates a digital signature in the Sigcryption phase, and the receiver decrypts and verifies the signature in the UnSigcryption phase. First, the public parameters of the system are generated, and the two participants independently generate their public keys and private keys. Then, the sender can preprocess the encryption operations with higher computational complexity in advance and send them to the receiver. Specifically, a secret random number is generated, and partial signature is generated using the secret random number. Then, the one-time public key generation algorithm OneTimePK is used to generate a one-time public key with the receiver’s public key and partial signature as input. The sender uses the one-time public key to encrypt the data to generate ciphertext, and sends the ciphertext and partial signature to the receiver, thereby completing data encryption and transmission preprocessing. After receiving the ciphertext, the receiver cannot decrypt the ciphertext and needs to authenticate the sender and apply for authorization. The sender uses the authorization algorithm Auth to generate an authorization value and sends it to the receiver. Finally, after the receiver obtains the authorization value, it uses the authorization value and its private key to generate a one-time private key through the one-time private key generation algorithm OneTimeSK and decrypts it. At the same time, the partial signature and the authorization value together constitute the digital signature of this data. The receiver can verify the integrity and source of the data through the verification algorithm Vertify.

The authorizable and pre-processable data transmission scheme is formally defined as follows:

*SysGen.* The system parameter generation algorithm $\textsf{SysGen}$ takes the security parameter $\lambda$ as input and generates the system’s public parameter *SP*

$$\begin{aligned} SP \leftarrow \textsf{SysGen}(\lambda ). \end{aligned}$$

*KeyGen.* Each participant *i* independently runs the key generation algorithm $\textsf{KeyGen}$, taking the system parameter *SP* as input and outputting their public and private key pair $(PK_i, sk_i)$

$$\begin{aligned} (PK_i, sk_i) \leftarrow \textsf{KeyGen}(SP), \quad i = 1, 2. \end{aligned}$$

Here, $i = 1, 2$ represent the two participants, i.e., the data sender and the data receiver.

*PreSign.* The sender runs the partial signature algorithm $\textsf{PreSign}$, taking the secret random number *r* and the system public parameter *SP* as input and outputting the partial signature *R*

$$\begin{aligned} R \leftarrow \textsf{PreSign}(SP, r). \end{aligned}$$

*OneTimePK.* The sender runs the one-time public key generation algorithm $\textsf{OneTimePK}$, taking the public key $PK_1$, the partial signature *R*, the data to be sent *msg*, and the receiver’s public key $PK_2$ as input and outputting the receiver’s one-time public key $PK_{2}^{OneTime}$

$$\begin{aligned} PK_{2}^{OneTime} \leftarrow \textsf{OneTimePK}(PK_1, R, msg, PK_2). \end{aligned}$$

*AsynEnc.* The sender runs the encryption algorithm $\textsf{AsynEnc}$, taking the receiver’s one-time public key $PK_2^{OneTime}$ to asymmetrically encrypt the data *msg*, outputs the ciphertext *C* and transmits it to the receiver, thus achieving preprocessing of secure data transmission

$$\begin{aligned} C\leftarrow \textsf{AsynEnc}(PK_{2}^{OneTime},msg). \end{aligned}$$

*Auth.* The sender runs the authorization algorithm $\textsf{Auth}$,inputs its private key $sk_1$, secret random number *r*, data *msg* and partial signature *R*, and outputs the authorization value *s* to achieve authorized data security transmission

$$\begin{aligned} s \leftarrow \textsf{Auth}(sk_1, r, msg, R). \end{aligned}$$

The partial signature *R* and the authorization value *s* constitute the final digital signature $\sigma \leftarrow (R, s)$ for the data *msg*.

*OneTimeSK.* The receiver runs the one-time private key generation algorithm $\textsf{OneTimeSK}$, taking their private key $sk_2$ and the authorization value *s* as input and outputting the one-time private key $sk_{2}^{OneTime}$

$$\begin{aligned} sk_{2}^{OneTime} \leftarrow \textsf{OneTimeSK}(sk_2, s). \end{aligned}$$

*AsynDec.* Upon receiving the ciphertext *C*, the receiver runs the decryption algorithm $\textsf{AsynDec}$, taking the system parameters *SP*, the one-time private key $sk_{2}^{OneTime}$, and the ciphertext *C* as input and outputting the plaintext *msg* or the termination symbol $\bot$

$$\begin{aligned} \begin{aligned}&msg/\bot \leftarrow \textsf{AsynDec}(SP,sk_{2}^{OneTime},C).\\ \end{aligned} \end{aligned}$$

*Verify.* The receiver runs the verification algorithm $\textsf{Verify}$, taking the sender’s public key $PK_1$, the data *msg* and the digital signature $\sigma$ as input, and outputting the validity judgment of the digital signature

$$\begin{aligned} Valid / Invalid \leftarrow \textsf{Verify}(PK_1, \textsf{hash}(msg, R), s). \end{aligned}$$

### Consistency

Given any $(SP, PK_1, sk_1, PK_2, sk_2, msg, C, \sigma )$, if the ciphertext *C* is a valid encryption of the data *msg* using the public key $PK_2$, and $\sigma$ is a digital signature of the data *msg*, and the decryption algorithm $\textsf{AsynDec}$ can return *msg* with overwhelming probability, and the verification algorithm $\textsf{Verify}$ can output Valid with overwhelming probability, then the scheme is said to have consistency

$$\begin{aligned} Pr\left[ \begin{array}{c} SP\leftarrow \textsf{SysGen}(\lambda ),\\ (PK_1,sk_1),(PK_2,sk_2)\leftarrow \textsf{KeyGen}(SP),\\ R\leftarrow \textsf{PreSign}(r,SP), PK_{2}^{OneTime}\leftarrow \textsf{OneTimePK}(PK_1,R,msg,PK_2),\\ C\leftarrow \textsf{AsynEnc}(PK_{2}^{OneTime},msg),\\ s\leftarrow \textsf{Auth}(sk_1,r,msg,R),\\ sk_{2}^{OneTime}\leftarrow \textsf{OneTimeSK}(sk_2,s),\\ msg'\leftarrow \textsf{AsynDec}(SP,sk_{2}^{OneTime},C),\\ Valid\leftarrow \textsf{Verify}(PK_1,\textsf{hash}(msg,R),s) \wedge msg=msg'. \end{array} \right] =1 \end{aligned}$$

### Confidentiality

Given any $(SP, PK_1, sk_1, PK_2, sk_2, msg, C, \sigma )$, if *C* is a valid encryption of the data *msg* using the public key $PK_2$, such that an attacker cannot derive any information about the original data *msg* from the ciphertext *C*, then the encryption algorithm is said to satisfy confidentiality

$$\begin{aligned} Pr\left[ \begin{array}{c} SP\leftarrow \textsf{SysGen}(\lambda ),\\ (PK_1,sk_1),(PK_2,sk_2)\leftarrow \textsf{KeyGen}(SP),\\ R\leftarrow \textsf{PreSign}(r,SP), PK_{2}^{OneTime}\leftarrow \textsf{OneTimePK}(PK_1,R,msg,PK_2),\\ C\leftarrow \textsf{AsynEnc}(PK_{2}^{OneTime},msg,\sigma ). \end{array} \right] =1 \end{aligned}$$

### Unforgeability of authorization

Given any $(SP, PK_1, sk_1, PK_2, sk_2, msg, C, \sigma )$, since the receiver obtains the authorization value *s* from part of the digital signature $\sigma$, if $\sigma$ is a valid signature on *msg*, then the receiver should be able to derive the authorization value *s*. If the verification algorithm $\textsf{Verify}$ can output Valid with overwhelming probability, then the scheme is said to have unforgeability of authorization

$$\begin{aligned} Pr\left[ \begin{array}{c} SP\leftarrow \textsf{SysGen}(\lambda ),\\ (PK_1,sk_1),(PK_2,sk_2)\leftarrow \textsf{KeyGen}(SP),\\ R\leftarrow \textsf{PreSign}(r,SP),\\ s\leftarrow \textsf{Auth}(sk_1,r,msg,R),\\ Valid\leftarrow \textsf{Verify}(PK_1,\textsf{hash}(msg,R),s). \end{array} \right] =1 \end{aligned}$$

## Scheme construction

### Complexity assumptions

The following are definitions of different difficult problems:

*Elliptic Curve discrete logarithm (DL) problem:* Given two group elements $G, H \in \mathbb {G}$, finding an integer $n \in Z$ such that $H = n \cdot G$ at any time is computationally infeasible.

*Computational Diffie-Hellman (CDH) assumption:* Given three group elements $G, a \cdot G, b \cdot G \in \mathbb {G}$, where $a, b \in Z$, any polynomial-time algorithm can only find an element $H \in \mathbb {G}$ with negligible probability such that the equation $H = ab \cdot G$ holds.

*Decisional Diffie-Hellman (DDH) assumption:* Given $G, a \cdot G, b \cdot G, c \cdot G \in \mathbb {G}$, any polynomial-time algorithm can only output the correct decision with negligible probability: if $c = a \cdot b$, the output is yes; otherwise, the output is no. If the output is yes, then the quadruple $(G, a \cdot G, b \cdot G, c \cdot G)$ is a DDH problem instance.

### Specific construction

The specific construction of the authorizable and preprocessable data transmission scheme is based on the SM2 encryption algorithm and Schnorr digital signature. Other encryption algorithms such as ECIES can also be used.

*SysGen:* The system parameter generation algorithm $\textsf{SysGen}$ is based on the elliptic curve $y^2=x^3+ax+b$ over a finite field $F_q$, where $a,b\in F_q$, and $4a^3+27b^2\ \textrm{mod}\ n \ne 0$. The generator of the elliptic curve group $\mathbb {G}$ is *G*, with order being a large prime number *n*, where $n>2^{191}$. The hash function $\textsf{hash}:\left( 0,1\right) ^* \rightarrow Z_n$ is defined, and the system public parameters are

$$\begin{aligned} SP=(a,b,\mathbb {G},G,n,\textsf{hash}). \end{aligned}$$

*KeyGen:* In the key generation algorithm $\textsf{KeyGen}$, the sender uses the system public parameters *SP* as input, selects a secret random number $\alpha _1 \in Z_n$, and computes

$$\begin{aligned} G_1:=\alpha _1 \cdot G \in \mathbb {G}. \end{aligned}$$

Thus, the sender’s private key is $sk_1=\alpha _1$, and the public key is $PK_1=G_1$. Similarly, the receiver’s private key is $sk_2=\alpha _2$, and the public key is $PK_2=G_2$, where $G_2=\alpha _2 \cdot G$.

*PreSign:* In the partial signature algorithm $\textsf{PreSign}$, the sender selects a random number $r \in Z_{n}^{*}$, uses the system public parameters *SP* as input, and calculates the partial signature *R*

$$\begin{aligned} R:=r \cdot G \in \mathbb {G}. \end{aligned}$$

*OneTimePK:* In the one-time public key generation algorithm $\textsf{OneTimePK}$, the sender uses the public key $PK_1=G_1$, the partial signature *R*, the data $msg \in \{0,1\}^*$, and the receiver’s public key $PK_2=G_2$ as input to compute the one-time public key $PK_{2}^{OneTime}$

$$\begin{aligned} \begin{aligned}&S:= R+\textsf{hash}(msg,R) \cdot PK_1,\\&PK_{2}^{OneTime}:=PK_2+S. \end{aligned} \end{aligned}$$

The sender will use the one-time public key $PK_{2}^{OneTime}$ to encrypt the data.

*AysnEnc:* In the encryption algorithm $\textsf{AysnEnc}$, the sender uses the system public parameters *SP*, the secret random number $k \in Z_{n}^{*}$, the data *msg*, and the one-time public key $PK_{2}^{OneTime}$ as input, and then computes as follows

$$\begin{aligned} \begin{aligned}&C_1:=k\cdot G\in \mathbb {G},\\&(x_1,y_1):=k\cdot PK_{2}^{OneTime} \in \mathbb {G},\\&t:=\textsf{KDF}((x_1,y_1),klen),\\&C_2:=msg\oplus t,\\&C_3:=\textsf{hash}(x_1,msg,y_1). \end{aligned} \end{aligned}$$

here, $\textsf{KDF}$ is a key derivation function that generates a random number of any length to be used as the symmetric encryption key. The specific construction of $\textsf{KDF}$ is based on the SM2 encryption algorithm; *klen* is the bit length of the data *M*. The ciphertext is $C = (C_1, C_2, C_3)$, which is sent to the receiver to complete the data transmission preprocessing.

*Auth:* In the authorization algorithm $\textsf{Auth}$, the sender uses the secret random number $r \in Z_{n}^{*}$, private key $sk_1=\alpha _1$, the data *msg*, and partial signature *R* as input to calculate the authorization value *s*

$$\begin{aligned} s:=r+sk_1 \textsf{hash}(msg,R). \end{aligned}$$

The authorization value *s* can be used by the sender to control the decryption time of the receiver, realize the authorization of secure data transmission, and form a digital signature $\sigma =(R,s)$ with the partial signature *R*.

*OneTimeSK:* In the one-time private key generation algorithm $\textsf{OneTimeSK}$, the receiver inputs the private key $sk_2 = \alpha _2$ and the authorization value *s*, and computes the one-time private key $sk_{2}^{OneTime}$ as follows

$$\begin{aligned} sk_{2}^{OneTime} := sk_2 + s. \end{aligned}$$

The receiver can use the one-time private key $sk_{2}^{OneTime}$ to decrypt the ciphertext *C*.

*AysnDec:* In the decryption algorithm $\textsf{AysnDec}$, the receiver inputs the public parameters of the system *SP*, the one-time private key $sk_{2}^{OneTime}$, and the ciphertext $C=(C_1,C_2,C_3)$. First, the receiver retrieves the partial ciphertext $C_1$ and checks if it is a point on the elliptic curve. If it is not, the process is terminated. Otherwise, the following computations are performed

$$\begin{aligned} \begin{aligned}&(x_2,y_2):=sk_{2}^{OneTime}\cdot C_1,\\&t:=\textsf{KDF}((x_2,y_2),klen), \end{aligned} \end{aligned}$$

If the value of *t* is all zeros, the process is terminated. Otherwise, the receiver retrieves the partial ciphertext $C_2$ and computes

$$\begin{aligned} M:=C_2\oplus t, \end{aligned}$$

then retrieves the partial ciphertext $C_3$ and computes

$$\begin{aligned} u:=\textsf{hash}(x_2,msg,y_2). \end{aligned}$$

The receiver then verifies if $u=C_3$. If not, the process is terminated; otherwise, the decrypted message *msg* is valid.

*Verify:* In the verification algorithm $\textsf{Verify}$, the receiver inputs the system’s public parameters *SP*, the sender’s public key $PK_1=G_1$, the data *msg*, and the digital signature $\sigma =(R,s)$. The receiver computes $v:=\textsf{hash}(msg,R)$ and verifies the equation

$$\begin{aligned} s\cdot G=R+v\cdot PK_1. \end{aligned}$$

If the equation does not hold, the verification is terminated. If the equation holds, the data *msg* is considered to be authentic and from the correct source.

## Application scenario

This scheme has been successfully applied to the scientific data zone system construction project of Beijing Academy of Science and Technology. In order to better protect and manage the scientific data assets of the unit, Beijing Academy of Science and Technology has built a data zone covering six major fields such as smart cities, life and health, ecological environment, and science and technology think tanks. The system brings together about 135 million high-value scientific research data. The Table 2 lists some specific examples of scientific data. Within the unit, different internal organizations can share and collaboratively use scientific data through the data zone system. In order to fully ensure the security of large amounts of data during sharing and circulation, in addition to taking traditional network security protection measures, the system also introduces the authorized and pre-processed data transmission solution proposed in this article, which effectively improves the confidentiality and integrity of scientific data during transmission, and ensures that data flows reliably and securely between different departments.

**Table 2:** Scientific data zone overview

Major domain	Application description	Data volume	Data type	File format
Environmental Protection	Includes information on soil, atmosphere, water bodies, environmental noise, pollutant emissions, etc., used in environmental pollution monitoring, management, evaluation, early warning, and more	Approx. 96.94 million entries	Text, structured data	txt, sql, xls, xlsx, excel
Urban Safety	Includes information on water supply and drainage, hazardous chemicals, traffic flow, safety incidents, etc., used for urban safety operation monitoring, simulation, evaluation, and disaster response	Approx. 11.376 million entries	Text, structured data	csv, txt, xlsx, excel, ora
Media Reports	Includes international media news report data, used in data mining, database construction, and scientific research	Approx. 15.00 million entries	Text	txt
Medicine and Health	Includes detection data on human organs, cells, drugs, as well as metabolic and chronic disease monitoring, used for drug development, chronic disease prevention, health management, testing, etc.	Approx. 6.297 million entries	Structured data, protein structure files	csv, pdf, txt, vcf, pdbqt

Through the application of this scheme, the scientific data zone has achieved efficient and secure data sharing among the internal institutions of the Beijing Academy of Science and Technology. A key management module is deployed in the system to uniformly maintain the public keys of each internal institution, while the private keys of each internal institution are securely stored locally. In response to the efficiency challenges brought by large-scale data security transmission, a two-stage mechanism of ”pre-processable and authorizable” is introduced: First, the data zone system performs a high-complexity pre-encryption operation on the data according to the data category, generates ciphertext and local signature, and stores them securely locally. This pre-processing stage reduces the burden of high-complexity encryption operations and improves the overall response efficiency of the system. When an internal institution needs to access a specific category of scientific data, it can establish a secure communication channel with the data zone system and obtain an authorization value after authentication. The internal institution can dynamically generate a one-time decryption private key locally based on the authorization value for subsequent ciphertext decryption operations. Through this mechanism, the controllability, confidentiality and integrity of data transmission are effectively achieved.

Despite its security and efficiency advantages, our proposed scheme faces several challenges in practice.First, in terms of the security and scalability of key management, it is necessary to ensure the local secure storage of private keys of each internal organization while implementing a unified management and dynamic update mechanism for public keys to cope with complex scenarios such as organizational changes and key rotation; secondly, the correctness and verifiability of one-time private key derivation are crucial to the security of the protocol. How to ensure the integrity of the authorization value, prevent the reuse or forgery of private keys, and achieve verifiability without leaking sensitive information are key issues that need to be addressed; finally, in terms of the composability and docking capabilities of the protocol, the solution needs to be able to be flexibly integrated into the existing data management system, seamlessly collaborate with authentication, encryption storage and network security modules, and have good modular design and scalability to adapt to future system evolution and heterogeneous environment requirements.

## Security analysis

In the specific construction of the proposed scheme, if the sender encrypts the data truthfully and generates a digital signature, the receiver, upon obtaining the authorization and the authorization value from the sender, can generate the correct decryption key using the authorization value and also verify the correctness of the digital signature. Therefore, the proposed scheme satisfies consistency, confidentiality, and unforgeability of authorization.

### Theorem 1

(Consistency) *Authorization Verification Consistency: For all key pairs and all data, any authorization value generated by the sender using the authorization algorithm will be verified as valid by the receiver.*

*Data Decryption Consistency: For all key pairs and all data, any ciphertext generated by the sender using the encryption algorithm will be verified as valid by the receiver under the corresponding decryption key.*

### *Proof*

Authorization Verification Consistency: For the authorization value *s*, we can calculate

$$\begin{aligned} \begin{aligned} R+\textsf{hash}(msg,R)\cdot PK_1 = r\cdot G +sk_1\textsf{hash}(msg,R) \cdot G = (r + sk_1\textsf{hash}(msg,R))\cdot G = s\cdot G. \end{aligned} \end{aligned}$$

Data Decryption Consistency: For all ciphertexts *C* containing $C_1$, we can calculate

$$\begin{aligned} \begin{aligned} sk_{2}^{OneTime}\cdot C_1&=ksk_{2}^{OneTime}\cdot G =k(sk_2+s)\cdot G =k(sk_2+(r+sk_1\textsf{hash}(msg,R)))\cdot G \\&=k\cdot (PK_2+S) =k\cdot PK_{2}^{OneTime}. \end{aligned} \end{aligned}$$

$$\square$$

### Theorem 2

(Confidentiality) *For all key pairs and all data, any ciphertext generated by the sender using the encryption algorithm remains secure when intercepted by a third party, meaning the third party cannot derive any information about the original data from the ciphertext.*

### *Proof*

For all data, the sender uses the SM2 encryption algorithm to encrypt the data. An attacker can access the public parameters $SP = (a, b, \mathbb {G}, G, n, \textsf{hash})$, the participants’ public keys $PK_1 = \alpha _1 \cdot G$, $PK_2 = \alpha _2 \cdot G$, the one-time public key $PK_2^{\text {OneTime}}$, the signature (*R*, *s*), and the ciphertext $C = (C_1, C_2, C_3)$, where $C_1 = k \cdot G$, $C_2 = msg \oplus t$, and $C_3 = \textsf{hash}(x_1, msg, y_1)$. Based on the Computational Diffie-Hellman (CDH) assumption, although the attacker knows both *G* and $C_1 = k \cdot G$, they cannot derive the secret value *k*. Consequently, they are unable to compute the intermediate values $(x_1, y_1)$, nor the masking value *t*, which is required to recover the plaintext. As a result, the attacker cannot retrieve the original message *msg* from $C_2$. $\square$

### Theorem 3

(Unforgeability of Authorization) *Let *$\mathcal {H}$* be a random oracle. If the CDH problem is hard, then the proposed scheme can be proven secure under the model of unforgeability against adaptively chosen message attacks, with a reduction loss of* $L = q_H$ *, where *$q_H$* is the number of queries to the random oracle. *

### *Proof*

For all authorization values provided by the sender, they are bound to the sender’s private key. No attacker can forge authorization values as the sender does. The formal proof of unforgeability of authorization will be detailed in the next section. $\square$

### Security model

#### Definition 1

Given the public key *PK*, a Probabilistic Polynomial-Time (PPT) attacker, after obtaining the valid digital signature it desires, has a negligible probability of computing a valid signature for a new message $msg^{*}$. The formal definition of the Existential Unforgeability under Adaptive Chosen Message Attacks (EUF-CMA) security model is as follows:

*Initialization:* Assume *SP* is the system parameter, the challenger $\mathcal {C}$ executes the key generation algorithm to generate a key pair (*PK*, *sk*), and sends *PK* to the adversary $\mathcal {A}$, while the challenger $\mathcal {C}$ retains *sk* to answer the digital signature queries from the adversary $\mathcal {A}$.

*Query phase:* The adversary $\mathcal {A}$ adaptively selects any message $msg_i$ for signature queries. For the message submitted by the adversary $\mathcal {A}$, the challenger $\mathcal {C}$ generates its digital signature $\sigma _{msg_i}$ and sends it to the adversary $\mathcal {A}$.

*Forgery phase:* The adversary $\mathcal {A}$ returns a forged signature $\sigma _{msg^*}$ for some message $msg^{*}$. If $\sigma _{msg^*}$ is a valid signature for $msg^{*}$ and no signatures for $msg^{*}$ have been queried before, the adversary wins the game, successfully forging a digital signature. The advantage of the adversary can be denoted as

$$\begin{aligned} Adv_{S}^{EUF-CMA}\left( \mathcal {A} \right) = Pr\left[ \mathcal {A}~forges\left( msg^*, \sigma _{msg^*} \right) \right] . \end{aligned}$$

If there exists no adversary that can win the above game with advantage $\varepsilon$ within time *t* after making $q_s$ signature queries, then the scheme is $(t, q_s,\varepsilon )$-secure under the EUF-CMA security model.

### Security proof

#### *Proof*

Assume there exists an adversary $\mathcal {A}$ who, under the EUF-CMA security model, can break the proposed scheme with an advantage probability $\varepsilon$ within time *t*. Then, it is possible to construct a simulator $\mathcal {B}$ that can solve the CDH problem in polynomial time. Given an elliptic curve group $(\mathbb {G}, G, p)$, the simulator $\mathcal {B}$ calls $\mathcal {A}$ and proceeds as follows:

*Initialization:* Let the system public parameter be $SP=(\mathbb {G}, G, p)$. $\mathcal {H}$ is a random oracle controlled by the simulator $\mathcal {B}$. For a valid public key *PK* and message *msg* input by the adversary, the simulator $\mathcal {B}$ can simulate generating a digital signature

$$\begin{aligned} \sigma \leftarrow (R, s). \end{aligned}$$

*Query phase:* The adversary $\mathcal {A}$ makes hash queries. The simulator $\mathcal {B}$ maintains a hash list that records all queries and responses. Initially, this hash list is empty. Let $\gamma _i$ be the *i*-th query value. If $\gamma _i$ exists in the hash list, the simulator $\mathcal {B}$ sends the corresponding value from the hash list to the adversary $\mathcal {A}$. Otherwise, the simulator $\mathcal {B}$ samples an output $\iota$ from $Z_q$ and returns $\iota \leftarrow Z_q.$ The prover randomly selects responses and challenges $z, w \leftarrow Z_q$ and then derives the commitment $R := g^z \cdot PK^{-w}$. The prover uses the random oracle $\mathcal {H}$ to output *w* for the input (*R*, *msg*, *PK*):

$$\begin{aligned} w \leftarrow \mathcal {H}(R, msg, PK). \end{aligned}$$

Thus, the adversary inputs $\mathcal {H}(R, msg)$ and queries the random oracle for the output *w*. Finally, the simulator $\mathcal {B}$ outputs $\sigma = (R, z)$ as the signature for the message *msg*.

*Forgery phase:* If the adversary can forge a valid signature $(msg^*, \sigma ^* = (R^*, z^*))$ for the message $msg^*$, it can reuse the same randomness to forge another signature for $msg^*$. According to the forking lemma, the adversary will output $(msg^*, \sigma ^* = (R^*, \hat{z}^*))$ with non-negligible probability. Given that $g^x = PK$, the simulator $\mathcal {B}$ will output

$$\begin{aligned} x := \frac{z^* - \hat{z}^*}{w^* - \hat{w}^*}. \end{aligned}$$

Knowing that $z = r + w \cdot sk$, and given that $z^*$ and $\hat{z}^*$ are associated with the same $R^*$, it is clear that $sk = x$. Therefore, the adversary capable of forging digital signatures can be used as a subroutine for extracting discrete logarithms for any challenge value.

Hence, the data authorization and secure asynchronous transmission scheme satisfies the EUF-CMA security model during the encryption process, ensuring that the authorization value generated by the sender is unforgeable. Additionally, the use of the SM2 encryption algorithm in this scheme also provides security against indistinguishability under chosen ciphertext attacks (IND-CCA). $\square$

## Comparison

**Table 3:** Comparative analysis of functions of different schemes.

Scheme	Data confidentiality	Data integrity	Preprocessable	Authorization correlation	Authorization unforgeability
S^14^	$\surd$	$\surd$	–	–	–
S^11^	$\surd$	$\surd$	–	–	–
S^24^	$\surd$	$\surd$	–	–	–
Ours	$\surd$	$\surd$	$\surd$	$\surd$	$\surd$

Table 3 presents a functional comparison of the proposed data security transmission scheme with other schemes^11,14,24^. Like other data security transmission schemes, the proposed scheme ensures Data Confidentiality and Data Integrity. However, this scheme uses an asynchronous processing method for encryption and digital signature operations, allowing the sender to complete encryption operations in advance, thereby enhancing processing efficiency. Additionally, the scheme integrates an authorization method during asynchronous data transmission, which not only secures the data transmission but also enhances the sender’s control over data access. As a result, the proposed scheme offers additional features such as Preprocessing , Authorization Correlation, and Authorization Unforgeability. These features are described below in detail:

*Data confidentiality:* The proposed scheme uses the SM2 encryption algorithm to ensure the confidentiality of the original data. Even if the encrypted data is intercepted by a third party, it remains impossible for them to derive the original data from the ciphertext.

*Data integrity:* The scheme uses the Schnorr digital signature algorithm, ensuring that the receiver can verify the integrity of the data. If a third party tampers with the original content, the verification algorithm will fail, indicating that the data is invalid.

*Preprocessable:* Through preprocessing, the sender can complete complex encryption operations ahead of time in a resource-unconstrained environment and send the ciphertext to the receiver. This preprocessing reduces the demand for computational resources and enhances overall system performance.

*Authorization correlation:* Before decryption, the receiver must request authorization from the sender. This mechanism ensures that only users who receive the authorization value can decrypt the data. The authorization value is uniquely associated with the receiver, preventing the misuse of authorization values between different receiver.

*Authorization unforgeability:* The authorization value generated by the sender is tied to their private key. Since the sender’s private key cannot be forged, the authorization value is also resistant to forgery, ensuring the security of the authorization process.

### Experimental analysis

The experiments were implemented using the Java programming language and the BouncyCastle library^1^. The experimental hardware environment included an x86_64 kernel operating system Windows, an Intel(R) Core(TM) i7-8750H CPU, and 16GB of memory.

For the 1KB file, the algorithm execution time of the scheme proposed in this paper is shown in Table 4.It is evident from the table that the execution time of the encryption algorithm was significantly higher than that of other algorithms. Therefore, in the proposed scheme, the sender can preprocess the encryption operation in advance and send the ciphertext to the receiver. When the receiver want to decrypt the data, they only need to spend minimal time obtaining the authorization value. In this way, the time required for the synchronous execution of the original Signcryption and Unsigncryption stages (28.37+0.58+3.32+4.58=36.85ms) is reduced to the time for the two-stage asynchronous execution (0.58+3.32+4.58=8.48ms).

**Table 4:** Execution time of different algorithms in scheme.

Algorithm	PreSign	OneTimePK	Auth	AsynEnc	OneTimeSK	AsynDec	Verify
Time (ms)	$\approx 1.91$	$\approx 3.34$	$\approx 28.37$	$\approx 0.58$	$\approx 0.008$	$\approx 3.32$	$\approx 4.58$

we also compared and analyzes the computational costs of the Signcryption phase (corresponding to the encryption algorithm of this paper’s scheme) and the Unsigncryption phase (corresponding to the decryption algorithm and signature verification algorithm of this paper’s scheme) in different schemes based on the overall computational cost of the scheme.In the comparison process, some insignificant operations in the scheme, such as XOR operations and Integer addition/multiplication operations, were ignored in order to more accurately evaluate the main computational costs of the scheme. Table 5 present the execution times for common operations used in schemes within the experimental environment. The system parameters were generated using the Secp160r1 elliptic curve, with the data size, hash value size, and random number size all set to 256 bits. The comparative analysis of different schemes’ execution times provided insights into their computational and communication costs in real-world applications.

**Table 5:** Execution time of cryptographic operations.

Notation	Function	Milliseconds (ms)
$T_e$	Modular exponentiation	$\approx 15.53$
$T_b$	Bilinear pairing operation	$\approx 30.03$
$T_{eccm}$	ECC point multiplication	$\approx 13.05$
$T_{ecca}$	ECC point addition	$\approx 2.02$

Table 6 shows the computational costs for the Signcryption and Unsigncryption phases across various data transmission schemes. Since the proposed scheme allowed for preprocessing the Signcryption phase locally, the overall computational cost for secure data transmission is reduced to $3T_{eccm} + T_{ecca} \approx 41.17ms$, which is significantly lower than other schemes^11,14,24^. For the communication overhead during data transmission, the proposed scheme, as well as the schemes in schemes^11,24^, are built based on elliptic curves. Hence, the element sizes in group $\mathbb {G}$ and integer domain $Z_q^*$ are set to 256 bits (32 B). The scheme in scheme^11^, built on bilinear pairing, set the element sizes in groups $\mathbb {G}_1$ and $\mathbb {G}_T$ to 512 bits (64 B). The scheme in scheme^14^, constructed using the RSA algorithm, uses an element size of 2048 bits (256 B) in integer domain $Z_q^*$ to maintain equivalent security levels. The original message size is 256 bits (32 B). Based on these settings, as shown in Table 6, we calculate the communication overhead of schemes^11,14,24^, and compare them to our scheme. Since the proposed scheme allowed the sender to locally preprocess the encryption operation and send the ciphertext to the receiver in advance, the communication overhead of the Signcryption phase could be considered negligible. The final communication cost was solely the size of the authorization value *s*, which is $|Z_q^*|=32B$, resulting in a lower communication overhead.

**Table 6:** Computation and communication costs comparison.

Scheme	Signcryption (ms)	Unsigncryption (ms)	Communication costs (Bytes)
S^14^	$2T_e\approx 31.06$	$6T_e\approx 93.18$	$| msg|+4| Z_{q}^{*}|=1056$
S^11^	$2T_{eccm}+2T_e\approx 57.16$	$2T_b+T_e\approx 75.59$	$|msg|+2|\mathbb {G}_1|+|\mathbb {G}_T|=160$?
S^24^	$3T_{eccm}+2T_{ecca} \approx 43.19$	$5T_{eccm}+3T_{ecca}\approx 71.31$	$| msg|+| \mathbb {G} |+2|Z{_q^*}|=128$
Ours	−	$3T_{eccm}+T_{ecca}\approx 41.17$	$| Z{_q^*}|=32$

Efficiency tests were conducted on this scheme with different file sizes (50KB-1600KB). Comparing the sender’s execution times with and without preprocessing demonstrates that the preprocessing operation greatly improves the efficiency of data transmission.

**Fig. 2:**
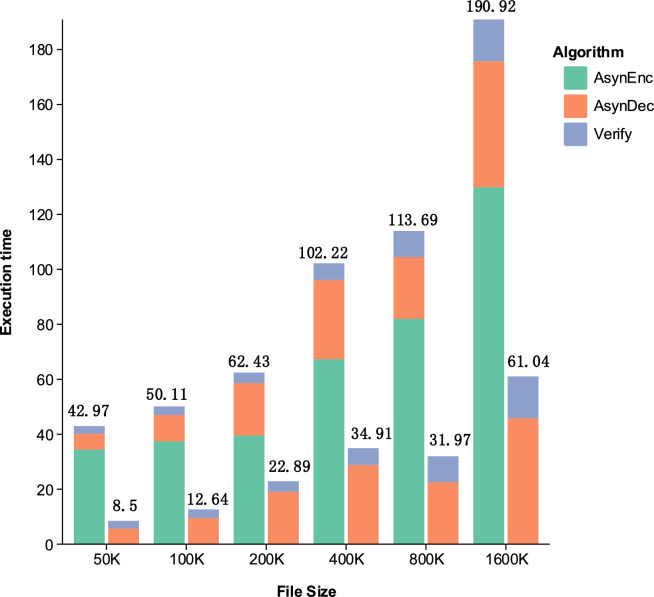
Efficiency comparison: preprocessing impact across file sizes.

As shown in Fig. 2, the proposed preprocessing method transforms the sequential execution time of encryption and digital signature (AsynEnc+AsynDec+Verify) into asynchronous execution time (AsynDec+Verify). With increasing file sizes, the preprocessing operation significantly enhances overall efficiency. For instance, at a file size of 50K, preprocessing improves efficiency by 80%, and even at 1600K, the efficiency still improves by 68%. This indicates that the preprocessing method is highly effective for ensuring secure data transmission, especially with large files. Moreover, despite the exponential increase in file size, the overall execution time remains relatively stable when using the asynchronous preprocessing method. This demonstrates that the proposed scheme is highly efficient and meets practical application requirements.

## Conclusion

This paper proposes an authorizable and preprocessable data security transmission scheme. The sender generates a one-time public key based on the local signature, public key and receiver’s public key, and then uses the one-time public key to encrypt the data and send it to the receiver to complete the preprocessing stage of the transmitted data. By asynchronously processing the encryption and digital signature in the general signcryption scheme, the efficiency of data transmission is improved. At the same time, the authorization mechanism is introduced to achieve secure data transmission, which not only ensures that the data cannot be abused, but also enhances the sender’s control over the data.Strict security proof shows that the scheme proposed in this paper meets EUF-CMA security.By comparing other signcryption schemes using different mechanisms, the results show that the use of a preprocessable method to complete the encryption operation in advance can greatly reduce the computational cost of data transmission. Experimental test results show that the efficiency of the scheme proposed in this paper can be improved by about 68%.

## Data Availability

The datasets generated and/or analysed during the current study are not publicly available due to Data Copyright but are available from the corresponding author on reasonable request.

## Footnotes

1 BouncyCastle is a popular open-source cryptographic library that provides implementations of various encryption algorithms and security protocols.
